# Development and Evaluation of Single Domain Antibodies for Vaccinia and the L1 Antigen

**DOI:** 10.1371/journal.pone.0106263

**Published:** 2014-09-11

**Authors:** Scott A. Walper, Jinny L. Liu, Daniel Zabetakis, George P. Anderson, Ellen R. Goldman

**Affiliations:** Naval Research Laboratory, Center for Bio/Molecular Science and Engineering, Washington, DC, United States of America; Weizmann Institute of Science, Israel

## Abstract

There is ongoing interest to develop high affinity, thermal stable recognition elements to replace conventional antibodies in biothreat detection assays. As part of this effort, single domain antibodies that target vaccinia virus were developed. Two llamas were immunized with killed viral particles followed by boosts with the recombinant membrane protein, L1, to stimulate the immune response for envelope and membrane proteins of the virus. The variable domains of the induced heavy chain antibodies were selected from M13 phage display libraries developed from isolated RNA. Selection via biopanning on the L1 antigen produced single domain antibodies that were specific and had affinities ranging from 4×10^−9^ M to 7.0×10^−10^ M, as determined by surface plasmon resonance. Several showed good ability to refold after heat denaturation. These L1-binding single domain antibodies, however, failed to recognize the killed vaccinia antigen. Useful vaccinia binding single domain antibodies were isolated by a second selection using the killed virus as the target. The virus binding single domain antibodies were incorporated in sandwich assays as both capture and tracer using the MAGPIX system yielding limits of detection down to 4×10^5^ pfu/ml, a four-fold improvement over the limit obtained using conventional antibodies. This work demonstrates the development of anti-vaccinia single domain antibodies and their incorporation into sandwich assays for viral detection. It also highlights the properties of high affinity and thermal stability that are hallmarks of single domain antibodies.

## Introduction

Vaccinia virus is the quintessential member of the *Poxviridae* family. It is a complex, enveloped virus that is characterized by a large dsDNA genome (∼190 kbp) that encodes approximately 250 genes [1]. The vaccinia virus is known for its use as a vaccine against its more pathogenic relative variola virus which is the causative agent of Smallpox. Though immunization with vaccinia virus is no longer conducted, the potential threat of Smallpox being used for biological attack and the infrequent outbreaks from the closely related Monkey pox virus warrants the need for continued research for vaccine development, detection assays, and therapeutics against members of the poxviridae family.

The L1 protein is a transmembrane, myristoylated protein that is located on the surface of the intact mature virion (IMV) [2], [3]. The 250-residue protein is an essential component of the viral life cycle as deletion mutants have been shown incapable of maturation [4]. The amino acid sequence of the L1 protein is highly conserved in vaccinia, variola, and the monkey pox viruses [2] and has been shown to be a target of antibodies that inhibit virus infection in plaque assays [5], [6].

Single domain antibodies (sdAbs) are the recombinantly produced variable domains of heavy chain only antibodies (HcAb) found in members of the family Camelidae [7], [8]. Replicating the antigen recognition domain of the parental HcAb structure, the sdAb binding pocket is formed from the loop-like complementarity determining regions (CDRs). Despite the reduced complexity of their structure sdAbs routinely demonstrate very high affinities (nanomolar) and specificities [9], [10]. Additionally, sdAbs frequently exhibit an ability to refold into an active recognition element following denaturation [11], [12]; in contrast to conventional antibodies and scFv that show a propensity to aggregate upon denaturation [13], [14], [15].

There is ongoing interest by the US Department of Defense to develop high affinity, thermal stable recognition elements to replace conventional antibodies in biothreat detection assays. As part of this effort, two llamas were immunized with a combination of killed vaccinia virus material and purified recombinant L1 antigen. It was hypothesized that this immunization protocol would lead to a diverse library that would allow for the isolation of sdAbs capable of binding the L1 antigen as displayed on the virus. In addition, as the animals were immunized with virus, the library would also yield sdAbs capable of recognizing a variety of viral antigens including envelope and membrane proteins of the viral particles. The isolated sdAbs could potentially find application in downstream assays and therapeutics.

## Materials and Methods

### Materials

Vaccinia virus Lister strain, sucrose gradient purified and certified killed by the UV-psoralen method [16] as well as vaccinia virus Lister strain certified killed by gamma irradiation were obtained from the Critical Reagents Program. Recombinantly produced vaccinia proteins, and anti-L1 monoclonal antibodies (mAbs) were obtained through BEI Resources and are listed in **Table 1**. The rabbit anti-vaccinia polyclonal antibody and anti- vaccinia mAb 3G4-1 were generously provided by Dr. Jill Czarnecki, Naval Medical Research Center.

**Table 1:** Recombinant vaccinia virus proteins and antibodies from BEI resources.

Protein	Product number	Citation
A27L	NR-2622	The reagent was obtained through the NIH Biodefense and Emerging Infections Research Resources Repository, NIAID, NIH: Vaccinia Virus (WR) A27L Protein with C-terminal Histidine Tag, Recombinant from baculovirus, NR-2622
A33R	NR-2623	The reagent was obtained through BEI Resources, NIAID, NIH: Vaccinia Virus, Western Reserve, A33R Protein with C-Terminal Histidine Tag, Recombinant from baculovirus, NR-2623
B5R	NR-546	The reagent was obtained through the NIH Biodefense and Emerging Infections Research Resources Repository, NIAID, NIH: Vaccinia Virus (WR) B5R Protein with N-terminal Histidine Tag, Recombinant from baculovirus, NR-546
F9L	NR-2626	The reagent was obtained through the NIH Biodefense and Emerging Infections Research Resources Repository, NIAID, NIH: Vaccinia Virus (WR) F9L Protein with C-terminal Histidine Tag, Recombinant from baculovirus, NR-2626
L1	NR-2625	The reagent was obtained through BEI Resources, NIAID, NIH: Vaccinia Virus, Western Reserve, L1R Protein with C-Terminal Histidine Tag, Recombinant from baculovirus, NR-2625
mAb417	NR-417	The reagent was obtained through the NIH Biodefense and Emerging Infections Research Resources Repository, NIAID, NIH: Monoclonal Anti-Vaccinia Virus (WR) L1R Protein, Residues 1 to 185 (similar to VMC-2), (produced *in vitro*), NR-417
mAb418	NR-418	The reagent was obtained through the NIH Biodefense and Emerging Infectious Research Resource Repository, NIAID, NIH: Monoclonal Anti-Vaccinia Virus (WR) L1R Protein, Residues 1 to 185 (similar to VMC-3), (produced (*in vitro*), NR-418
mAb419	NR-419	The reagent was obtained through the NIH Biodefense and Emerging Infectious Research Resource Repository, NIAID, NIH: Monoclonal Anti-Vaccinia Virus (WR) L1R Protein, Residues 1 to 185 (similar to VMC-4), (produced (*in vitro*), NR-419

The XL1-Blue *Escherichia coli* strain (Agilent Technologies) was used for library construction and throughout the biopanning selection process. The *E. coli* strain DH5α was used for routine cloning experiments; *E. coli* Rosetta and Rosetta (DE3) strains (Merck Millipore) served as the bacterial strains for sdAb production.

Restriction enzymes, *Taq* DNA polymerase, and T4 DNA ligase for library construction and cloning were purchased from New England Biolabs (NEB). Mutagenesis was performed using the QuikChange II site-directed mutagenesis kit (Agilent Technologies) following the manufacturer instructions. All oligonucleotides were ordered through MWG Operon; DNA sequencing performed by MWG Operon. Anti-camel HRP was from Bethyl Laboratories. Chemical reagents were acquired primarily from Sigma Aldrich, Fisher Scientific, or VWR unless otherwise noted.

### Immunizations and library construction

Two llamas maintained by Triple J Farms (Bellingham, WA) were subjected to a series of identical immunizations with both killed vaccinia virus (UV-psoralen inactivated) and recombinant L1 antigen. The immunization cycle consisted of three rounds with killed virus material (100 µg, 100 µg, and 50 µg) and two additional rounds with the L1 proteins (100 µg each time). The immunization protocol used in this work was specifically approved by the Triple J Farms Institutional Animal Care and Use Committee (IACUC). Triple J Farms has an active IACUC that reviews and approves all immunization protocols. Plasma was titered to assess binding to both the killed vaccinia reagent and L1 following the third and fifth immunization.

Serum was separated from whole blood samples via low speed centrifugation (1000 x g) and the use of Uni-Sep Maxi+ conical tubes (Novamed). The buffy coat was transferred to new tubes and peripheral blood lymphocytes (PBLs) pelleted at 2500 x g in a Beckman clinical centrifuge. Total RNA was extracted from PBLs using the QIAGEN RNA Blood Mini Kit and manufacturer's protocol. Extracted total RNA was converted to a cDNA library using the Superscript II First Strand synthesis kit (Invitrogen) and manufacturer's protocol. The cDNA was subjected to two rounds of PCR, the first using degenerate primers for variable domain amplification and the second to incorporate directional SfiI restriction sites. To facilitate phage display, the sdAb library and pECAN 21 vector [15] were digested with SfiI and gel purified. To improve ligation efficiency the 5′ phosphate of the cleaved vector backbone was removed using Calf Intestinal Alkaline Phosphatase (NEB) and a subsequent column purification using the QIAGEN PCR Purification kit. Vector and insert DNA were quantitated using a Nanodrop 1000 (Thermo Scientific) and ligated at a 3∶1 (insert: vector) ratio overnight at 16°C with T4 DNA ligase (NEB). The ligation reactions were pooled and column purified as before and diluted to a final concentration of 100 ng/µl. Electroporation of XL1-Blue cells was accomplished using a MicroPulser Electroporation Apparatus (Bio Rad) and 0.1 cm electroporation cuvettes. Following recovery, cultures were plated to antibiotic containing media and grown overnight at 37°C. Individual clones from dilution plates were counted and sequenced to determine library size and diversity.

### Selection of target binding sdAbs

The sdAb library was interrogated against both recombinant L1 antigen and intact killed vaccinia virus. Antigen was immobilized to microtiter plates either passively or, in the case of the L1 antigen, captured by antibodies that were themselves passively immobilized (sandwich-style). Target antigens and antibodies were diluted in phosphate buffered saline (PBS, pH 7.4) and aliquoted to individual wells of Nunclon MaxiSorp microtiter plates overnight at 4°C. The viral material was immobilized at a concentration of 10 µg/ml. For the sandwich-style selection, each monoclonal antibody (mAb417 and mAb419) was immobilized individually to separate wells at a concentration of 3 µg/ml. L1 antigen concentrations of 3–5 µg/ml were utilized for both the direct immobilization and sandwich-style selections. After overnight incubation, wells were washed twice with PBS containing 0.05% Tween 20 (PBST) and then blocked with PBST plus 5% milk proteins (PBSTM).

Individual rounds of selection were carried out as previously described [17], [18], [19]. Briefly, phage from the initial library or amplified phage from preceding rounds were diluted in PBSTM to an approximate concentration of 1×10^12^ pfu/ml. Phage were incubated with antigen for 1 hour with agitation. Wells were washed with PBST, the number of washes increasing with each round of biopanning. Bound phage were eluted with 100 mM triethylamine then neutralized with an equivalent volume of 1 M Tris-HCl (pH 8.0). Approximately half of the eluted phage volume was then used to infect an early-log phase XL1-Blue culture. This culture was incubated for one hour at 37°C then plated to antibiotic containing media. Following an overnight incubation, colonies were dislodged from the solid media, a portion of which was used for phage amplification and the remainder diluted in sterile glycerol to a final concentration of 40% v/v for storage at −80°C. Amplified phage was used for additional rounds of selection.

Following three rounds of selection individual clones were chosen from output plates and assayed for target binding capabilities using a monoclonal phage ELISA [20]. A horseradish peroxidase (HRP) anti-M13 antibody served as the secondary antibody and the SigmaFast OPD substrate used for quantitation of binding events. Individual clones were considered positive if signal was at least threefold over background. A selection of clones that showed binding as judged from the ELISA, was sequenced to determine unique binders. MultAlin was used to align sequences for presentation [21].

### Protein expression and purification

The coding sequence of unique binders was mobilized to pECAN45 [19], [22] or pET22b (Merck Millipore) for protein expression. For expression in pET22b, sdAb genes were amplified from the phage display vector (pECAN21) using universal primers that anneal within the 5′ leader sequence (Ncoforward - ggcccagccggccatgg) and upstream of the hinge region of the sdAb (Notxhoback - gctcgagtgcggccgctgaggagacggtgacctgggtccc). The PCR product and the pET22b vector were digested with the NcoI and NotI restriction enzymes (NEB), gel purified, and then ligated at a 3∶1 insert: vector ratio using T4 DNA ligase (NEB). The nucleotide sequence of individual clones was confirmed prior to transformation to Rosetta (DE3) *E. coli* for protein expression. The sdAbs in the pECAN45 expression vector were transformed to Rosetta *E. coli* for protein expression. Expression and purification from 500 ml shake flasks was performed as previously described, with the exception that protein was eluted from the nickel sepharose resin using 0.5 M imidazole [15], [22], [23]. A Superdex G75 10/300GL column (GE Healthcare Services) and BioLogic DuoFlow Chromatography system (BioRad Laboratories) were used for FPLC purification following immobilized metal ion affinity chromatography. Protein concentration was determined using a Nanodrop 1000 spectrophotometer (ThermoFisher Scientific) and calculations based on absorbance at 280 nm. Several of the sdAbs including L1-G2, L1-H7, and Vacc-E7, were purified at least two separate times, yielding around 18–20 mg per liter of bacterial expression culture; the L1-G2+ mutant yielded between 3–4 mg per liter on replicate preparations.

### MAGPIX assays

Both direct and sandwich assays were performed to characterize the expressed sdAbs and conventional antibodies similar to assays described previously [24], [25]. Viral material and proteins were immobilized to Magplex beads covalently using EDC/NHS reagents and the standard two step protocol provided by the manufacturer. For direct binding assays, killed vaccinia virus material, L1 antigen, and the other recombinantly produced vaccinia proteins from BEI resources detailed in **Table 1** (A27L, A33R, B5R, and F9L) were immobilized to magnetic spheres at a saturating concentration of 100 µg/ml. We verified that the antigen was immobilized in an active form that could be recognized by antibodies by performing binding assays utilizing monoclonal and polyclonal antibodies (data not shown). For sandwich assays, conventional antibodies and sdAbs were immobilized at concentrations of at least 1 mg/ml and 100 µg/ml, respectively.

Purified sdAbs and conventional antibodies were biotinylated to serve as tracers in both direct and sandwich assays using a sulfo NHS –LC-LC- biotin (Pierce) reagent as previously described [17], [19]. Free biotin was removed using a BioGel P10 chromatography column and an EconoPump system (BioRad Laboratories) to monitor the absorbance at 280 nm for fraction collection. Conventional antibody tracers were used at a concentration of 10 µg/ml while the smaller sdAbs were used at concentrations of 1 µg/ml. A streptavidin conjugated phycoerythrin (Columbia Biosciences) was used as the reporter at a concentration of 5 µg/ml.

In the MAGPIX experiments, binding is measured from greater than 50 beads; the error of the mean fluorescence intensity (MFI) was found to be 5% or less. Each of the direct binding and sandwich assays were performed on at least two different days. In each case, the experimental data replicated. For some clones, protein from duplicate protein productions was both biotinylated and immobilized on bead sets. Detection trends observed in MAGPIX assays were also consistent across protein preparations; minor variations observed were likely due to variability in the biotinylation and immobilization of the sdAbs.

Sandwich assays were also used to screen phage-displayed sdAbs for antigen binding. The anti-L1 monoclonal antibodies 417 and 419 (**Table 1**) were used to capture L1. Phage were introduced and detected by biotinylated anti-M13 and streptavidin phycoerythrin.

### Sandwich and direct binding ELISA

Sandwich ELISAs were performed as described previously [19]. The sdAbs were passively immobilized in wells of 96-well plates at a concentration of 1 µg/ml. Wells were blocked in blocking buffer (PBSTM), and then L1 antigen was added at four fold dilutions from 1000 ng/ml, including a no antigen control. Biotinylated mAb and sdAb (at 1 µg/ml) were used as reporter reagents. Signal was developed using horseradish peroxidase conjugated streptavidin in conjunction with SigmaFast OPD substrate.

Direct binding ELISAs were performed essentially as described previously [18], [25]. Briefly, antigen (i.e. intact virions) was passively immobilized to a Nunclon microtiter plate. Following one hour incubation in blocking buffer, biotinylated sdAbs and conventional antibodies were diluted in blocking buffer to equivalent molar ratios then incubated with antigen for one hour. A horseradish peroxidase conjugated streptavidin served as the secondary reagent. The SigmaFast OPD substrate was used for analysis of binding as described above.

### Surface plasmon resonance

A BioRad ProteOn XPR36 system and standard GLC sensor chip was used to assess binding kinetics [17], [19]. The L1 antigen was immobilized to the sensor chip surface on four rows at saturation concentrations of 10 µg/ml using EDC/NHS chemistry. Protein was flowed over the activated surface for 800 s at a flow rate of 30 µl/min. The surface was inactivated with ethanolamine. For assay the chip was turned 90 degrees, then dilutions (100 nM to 0 nM) of each sdAb were flowed across the chip for 120 s at 100 µl/min and binding was recorded on the four L1-coated lanes; next, the buffer was flowed over for 600 s and the dissociation monitored. The surface was regenerated with 50 mM glycine (pH 2.5) between individual samples. The one shot kinetics were determined from each of the L1 coated rows using five concentrations of single domain antibody. Kinetic parameters were calculated using the ProteOn Manager RM 2.1 software (Bo Rad). The range of values from the four measurements was always within a factor of 2, and typically the four values were within 20%.

### Fluorescence based melting assay

The fluorescence based melting assay was conducted using an applied Biosystems Step One Real Time PCR system and Sypro Orange dye (Sigma) [26], [27]. A total of 10 µg of purified sdAbs was added to a 20 µl volume of PBS buffer. The Sypro Orange dye was diluted 1000-fold each into each reaction solution. The temperature was increased from 25 to 99°C at a rate of 1.2°C/min. All measurements were done in triplicate and the values agreed within 0.6°C. The fluorescence based melting measurements were also performed at least twice giving results that were essentially identical. For several sdAb clones, the melting temperature (Tm) of multiple protein preparations was measured by fluorescence based melting assay; the values determined from the different preparations agreed within a degree.

### Circular dichroism

Refolding and melting temperatures were assessed using a Jasco J-815 CD spectrometer [18], [25]. Samples were dialyzed overnight in deionized water then diluted to a final concentration of 40 µg/ml in water. The differential absorbance of the protein sample was measured at 208 nm to monitor the secondary structure of the protein as the temperature was increased from 25 to 90°C incrementally at a rate of 5°C/min. The melting point correlated to the temperature at the inflection point between the folded and unfolded state. The error on the T_m_ determinations is within ±1°C. Most samples were analyzed by two independent measurements. For three of the samples (L1-H7, L1-G2, and L1-G2+) replicate protein preparations were analyzed by CD. Replicate measurements showed essentially the same melting and refolding behavior.

### Thermostability assays

SdAbs and mAbs were diluted to 1.0 mg/ml concentration in PBS (pH 7.4). The samples were heated for 1 hour at 65°C, 70°C, 75°C, 80°C and 85°C in a Tetrad2 thermocycler (MJ Research). All samples were allowed to cool to room temperature on the bench top. Samples were centrifuged to pellet aggregated material then optical density was measured at 280 nm to determine protein concentration. Error on the solubility measurements, determined from duplicate experiments was ±5%. Remaining soluble protein was assayed for binding activity using SPR monitoring binding of the samples to four L1-coated rows. Binding activity was based on the SPR signal generated by 50 nM of sdAb or mAb, measured at 30 seconds, which is still near 1st order binding. Results were reported as percent activity; the error on this value was less than 10%.

## Results and Discussion

### Immunization and library construction

Two llamas (Whisper and Centavo) were subjected to five rounds of immunization with killed vaccinia virus (3 immunizations) and recombinant L1 antigen (2 immunizations). This immunization strategy was based on our previous work in developing sdAbs towards the MS2 phage [24]. In that work we found that sdAbs derived from llamas immunized only with recombinant coat protein from the MS2 phage were not able to recognize the intact phage in direct binding experiments; by immunizing first with phage and then with the recombinant coat protein we isolated high affinity binders that recognized both targets. We speculated that using a similar strategy would enable the isolation of sdAbs that were able to bind the L1 antigen as well as whole vaccinia virus.

Whole blood samples were collected from each animal after the three vaccinia immunizations and again after the complete series. The plasma was titered to examine binding towards both the killed vaccinia and L1 antigen (**Figure S1 in File S1**). After the vaccinia immunizations the animals showed a robust response against the killed viral material, but not the L1 antigen. The llama Centavo showed a higher titer than Whisper. After the full immunization course, both animals had improved recognition of L1; however the titer from Centavo was still much better than from Whisper.

Buffy coat fraction was isolated from the blood collected after the complete immunization protocol and served as the source of peripheral blood lymphocytes (PBLs) for library construction. Total RNA was extracted, converted to cDNA, and subjected to a two stage PCR to amplify the variable domain genes as described elsewhere [8], [15]. The sdAb genes and pECAN 21 vector were digested with SfiI, gel purified, ligated, and transformed into XL1-Blue cells via electroporation. The library size was calculated to be approximately 3.8×10^7^ based on direct plate counting and sequencing of a small sample of individual clones.

### Selection of L1 binders

Initial selection was conducted with passively immobilized recombinant L1 antigen. After three rounds of biopanning, individual clones were assayed for binding to target antigen using a monoclonal phage ELISA protocol. Although binding clones were identified by monoclonal phage ELISA, few of these showed strong binding to the recombinant L1 antigen in MAGPIX assays using the phage-displayed sdAbs (**Figure S2 in File S1**). Given that the animals were immunized with the recombinant protein as well as killed viral material it was anticipated that selection against this protein would have been more successful. It was therefore hypothesized that the L1 antigen may not be amenable to passive immobilization as was used during the initial rounds of selection. An alternate selection protocol was pursued in which the recombinant L1 antigen was captured by conventional antibodies for the L1 antigen. It was believed that this method would ensure that L1 antigen remained in its native conformation and enable the isolation of sdAbs better able to recognize the target antigen. Positive clones were sequenced then representative sdAb were cloned into an expression vector for subsequent purification, biotinylation, and characterization as previously described [17], [18], [19], [25].

**Figure 1:**
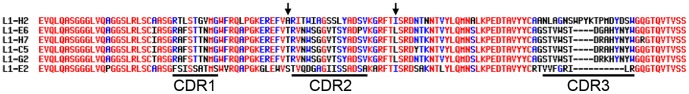
Sequences of L1 binding sdAb. The alignment shows the sequences of L1 binders isolated in sandwich-style selection. Three families, based on homology within the CDR regions were isolated. CDR regions are underlined. Red denotes positions where the amino acid is 90% conserved in the compared sequences, blue indicates low consensus (50%) and black are not conserved. The most dominant family (L1-H7 family) showed some variation in amino acid sequences of the framework regions that showed an impact of binding affinities and thermal stability. Arrows mark the positions of the cysteine substitutions in the L1-G2+ construct.

Three rounds of selection were conducted with the initial library using the monoclonal antibodies mAb417 and mAb419 as immobilized capture agents of the L1 antigen. These mAbs were chosen as they have been shown to bind different epitopes on the L1 antigen [5]; further information on the monoclonal antibodies is provided in **Table 1**. Clones identified through a combination of phage ELISA and MAGPIX assays were sequenced and fell into three distinct families: the L1-H7 family with the most members; L1-H2 family with 2 members and L1-E2 with one member. Members of each sequence family had only minor differences with essentially identical complementarity determining regions (CDRs). **Figure 1** shows the sequence of clones L1-H2, L1-H7, L1-E2, L1-G2, L1-C5, and L1-E6 that were mobilized into an expression vector for production as soluble sdAbs.

**Figure 2:**
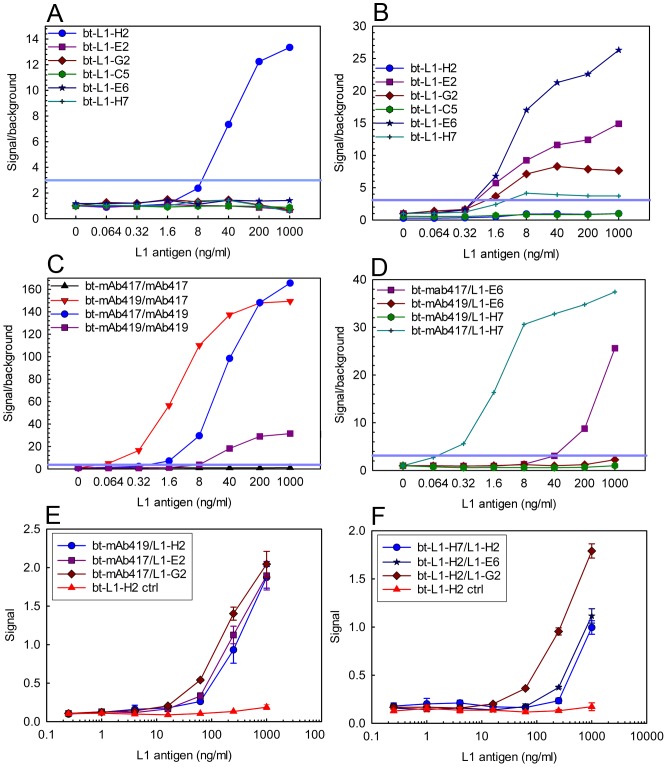
Sandwich immunoassay for L1 detection. These assays were performed on at least two different days; representative binding assays are shown. Panels A-D are MAGPIX assays and are plotted as signal over background versus the L1 concentration; a signal over background of 3 was set as the limit of detection and is indicated by a blue line. Each MAGPIX assays measures binding from at least 50 beads and the error of the mean fluorescence intensity is within 5%. Panel A, mAb419 capture with the biotinylated (bt)-sdAb tracers. Panel B, mAb417 capture with the bt-sdAb tracers. Panel C shows combinations of the mAb capture and tracer pairs. Panel D shows the sdAb L1-E6 and L1-H7 captures paired with the mAb reporters. Panels E and F are ELISA data, each point represents the average of three measurements and the error bars represent the standard deviation. Control lines were collected with the bt-L1-H2 tracer in combination with a non-L1 binding capture sdAb. Panel E shows sdAb capture and mAb reporters. Panel F shows sdAb capture and tracer pairs.

Following purification, the sdAbs were incorporated into sandwich assays which combined both sdAbs and mAbs to identify successful combinations for L1 detection, **Figure 2**. As seen in panels A and B of **Figure 2**, members of the L1-H7 family of sdAbs, and L1-E2 bound the same epitope as mAb mAb419 and could be used in combination with mAb417. We defined our limit of detection as a signal to noise ration of at least 3. The L1-H2 sdAb appeared to have a binding site which coincided with the epitope of mAb417, and could be used with mAb419 in sandwich assays. Interestingly, although L1-C5 is a member of the L1-H7 family, it did not function as a reporter when paired with mAb417; it showed only minor signal when paired with the L1-H2 capture with a signal to noise ratio of under 3 (data not shown). Although the sdAb could prove effective captures in MAGPIX assay, as shown in panel D of **Figure 2**, most did not function well when covalently immobilized on the Magplex beads. In addition to the MAGPIX sandwich assays, we demonstrated the ability of the L1-specific sdAbs to function as both reporters and captures in an ELISA format as shown by panels E and F of **Figure 2**. In both MAGPIX and ELISA, the best limits of detection were achieved when the sdAbs were paired with mAbs. Several options are available that could lead to more sensitive sandwich assays utilizing only sdAbs. Genetic constructs that facilitate directional immobilization have the potential to improve sdAb capture reagents [23]. Alternate approaches include improving the affinity of L1-H2 or screening the library for additional members of the L1-H2 family which may function better in sandwich assays.

### Determining binding kinetics using surface plasmon resonance (SPR)

The binding kinetics for each of the L1 binding sdAbs was determined using SPR. All seven of the L1 binders chosen for characterization showed low nanomolar or better affinities for the antigen, **Table 2** and **Figure S3 in File S1**.

**Table 2:** Binding kinetics for sdAbs targeting the L1 antigen*.

sdAb	k_a_ (1/Ms)	k_d_ (1/s)	K_D_ (M)
L1-C5	7.2×10^6^ (1.2×10^6^)	3.2×10^−2^ (3.9×10^−3^)	4.0×10^−9^ (2.1×10^−10^)
L1-E2	6.5×10^6^ (1.6×10^6^)	4.5×10^−3^ (1.0×10^−3^)	7.0×10^−10^ (2.4×10^−11^)
L1-H2	1.2×10^6^ (1.5×10^5^)	2.1×10^−3^ (1.7×10^−4^)	1.8×10^−9^ (9.0×10^−11^)
L1-E6	3.9×10^5^ (3.2×10^4^)	1.0×10^−3^ (3.0×10^−5^)	2.6×10^−9^ (1.2×10^−10^)
L1-H7	5.6×10^5^ (4.3×10^4^)	8.7×10^−4^ (8.4×10^−5^)	1.5×10^−9^ (3.8×10^−11^)
L1-G2	5.3×10^5^ (3.8×10^4^)	1.0×10^−3^ (5.4×10^−5^)	1.9×10^−9^ (2.0×10^−10^)
L1-G2+	2.1×10^6^ (5.0×10^5^)	2.1×10^−3^ (2.8×10^−4^)	1.0×10^−9^ (1.3×10^−10^)

* From binding to at least 3 L1-coated spots, reported as average (standard deviation).

The L1-H7 family, though highly conserved (97% identical), did contain some members with amino acid differences that appeared to directly contribute to the binding kinetics and thermal stability as seen with sdAb L1-C5. The sdAb L1-C5 showed an increase in the off-rate by two orders of magnitude compared to other members of the family; this clone has a single N to Y substitution in the framework 3 region that is not observed in any other members of the L1-H7 family. This shows how minimal changes in the framework can have a significant impact on binding affinity and stability.

### Melting temperature and thermal stability

The thermal stability of individual sdAbs was assayed using a combination of techniques. Initially, the melting temperature for each sdAb was determined using a Sypro Orange fluorescent based melting assay. Sypro Orange is quenched in an aqueous environment but fluoresces as it binds hydrophobic patches that are exposed as the protein unfolds. The L1 binders had a range of melting temperatures from 56–62°C, **Table 3**.

**Table 3:** Melting temperatures of L1 binding sdAbs and mAbs.

sdAb/mAb	Melting temperature (°C) (Fluorescence melt assay*)	Melting temperature (°C) (Circular dichroism**)
L1-C5	57	58
L1-E2	56	56
L1-E6	60	60
L1-G2	61	64
L1-G2 +	80	81
L1-H2	62	66
L1-H7	60	62
mAb 417	71	ND
mAb 418	71	ND
mAb 419	70	ND

ND  =  not determined.

* Fluorescent melt assays performed in triplicate on at least 2 days. The triplicate measurements were typically identical within less than 0.2°C, and always within 0.6°C, while samples measured from different protein lots or on different days agreed within a degree. ** Most of the CD assays were performed on at least two different days. Values determined by CD agreed within a degree.

**Figure 3:**
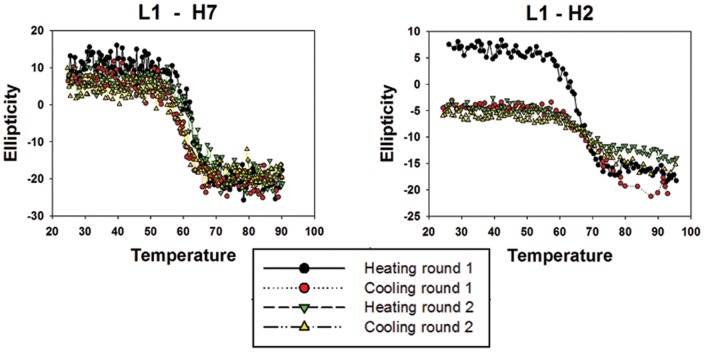
Refolding of sdAb. Circular dichroism was used to monitor the secondary structure of purified sdAbs over a temperature range of 25–95°C. Representative plots are shown of clones H7 and H2 typifying the distinctly different refolding properties. Temperature is expressed in °C and the change in ellipticity is in milidegrees. CD experiments were performed in duplicate for most of the sdAbs; the observed Tm values agreed within a degree, and the refolding trends were the same among the replicate measurements.

Most sdAbs demonstrate a propensity to refold following denaturation, one of the benefits of the low complexity structure [12], [15]. To assess the refolding capability circular dichroism (CD) was used to monitor the secondary structure of the L1 binders as the temperature of the solution was gradually increased up to 95°C and then decreased back to room temperature to facilitate unfolding and refolding. The CD analysis indicated that the members of the L1-H7 family were significantly more successful in regaining their native structure compared to L1-H2 or L1-E2, **Figure 3** and **Figure S4 in** **File S1**. Both L1-H2 and L1-E2 showed a dramatic decrease in ellipticity after the first round of heating and cooling. This decrease occurred only in the first cycle indicative of a failure to refold, however subsequent rounds showed no additional decrease in ellipticity suggesting that the portion of the sdAb preparation which refolded initially continued to refold successfully.

As was seen with the binding kinetics, the amino acid variations in the L1-H7 family contributed to distinct differences in the refolding properties of individual members. The sdAbs L1-H7 and L1-G2 were able to refold to near completion through several cycles of heating and cooling. In contrast, the L1-C5 and L1-E6 sdAbs exhibited a loss in ellipticity after the first heating cycle, an indication of failure to recover secondary structure during the cooling cycle. Subtle variations in amino acids sequences seem to have negative effects on the overall stability of these L1 binders.

**Figure 4:**
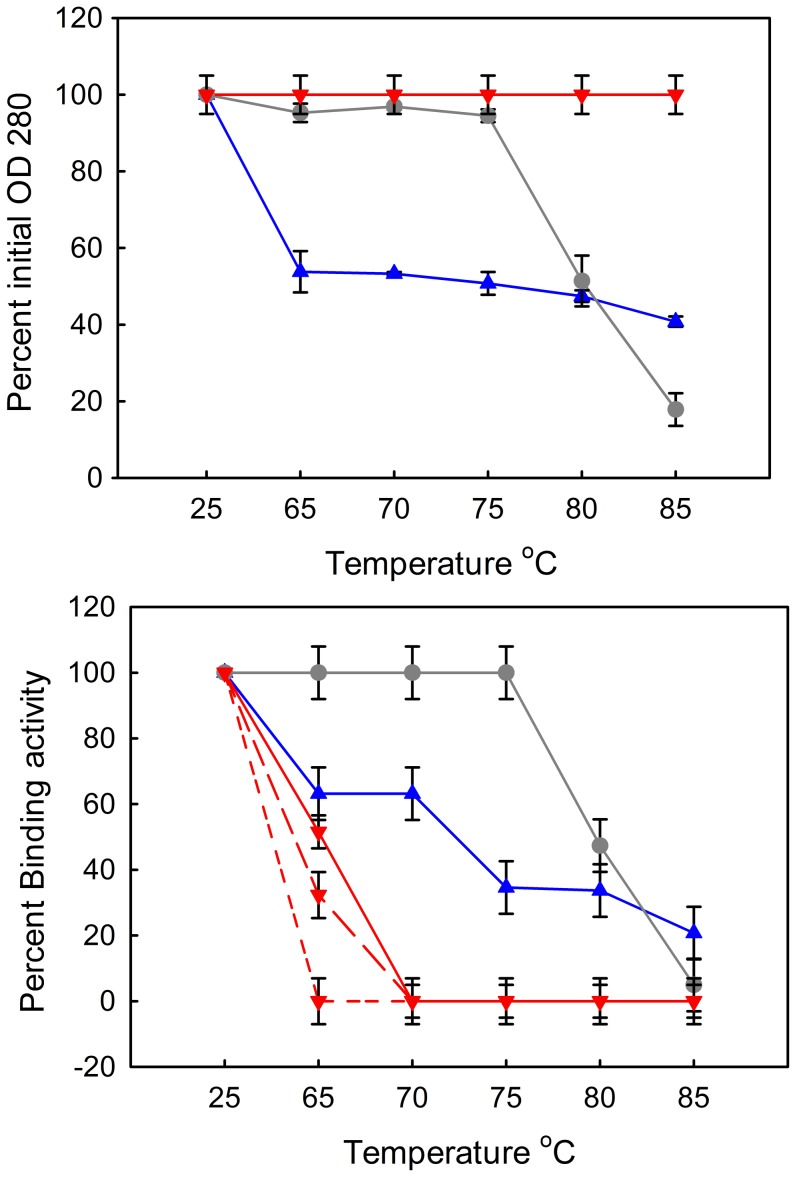
Thermal stability assay. Following a one hour incubation at the defined temperature optical density at 280 nm was measured to assess protein aggregation (upper panel). Samples G2 (blue triangles) and G2+ (gray circles) were measured in duplicate, and showed the same solubility behavior in each replicate. Since all of the mAbs behaved similarly they are all represented by the same symbol (red upside-down triangle). Binding activity following incubation was measured using a direct binding assay with the L1 antigen directly immobilized to four rows of a sensor chip for SPR analysis (lower panel). The mAbs have the same color and symbol but are differentiated by line type: mAb419 with short dashes, mAb418 with long dashes, and mAb417 with a solid line.

While CD is able to show the proteins losing and recovering secondary structure when heated at low concentration, it is important to assess the activity of sdAbs after heating to correlate refolding with antigen recognition. SdAbs and mAbs were heated, at a concentration of 1 mg/ml, to defined temperatures and incubated for 1 hour. The samples were allowed to cool to room temperature, and centrifuged to pellet precipitated protein prior to measuring the optical density. Although unfolded material and smaller aggregates may stay in solution, optical density measurements provide information on which samples contain a percentage of soluble protein. If the majority of protein precipitated then the sample would not be expected to show much in terms of binding activity. Samples that retained some soluble antibody were assayed for binding capabilities via SPR, **Figure 4**. Clones L1-H2, L1-E2, and L1-C5 which demonstrated a low propensity to refold in CD, aggregated extensively and essentially all the protein precipitated following incubation at temperatures above their melting point and were therefore not assayed for activity. Clone L1-E6 also appeared to aggregate, with only 10–20% of the protein left in solution after heating. In contrast, a substantial amount of L1-G2 remained in solution after heating (Figure 4). Clone L1-G2 showed continued binding activity following incubations at temperatures as high as 85°C, though significantly reduced at the highest temperatures tested. Interestingly, none of the mAbs showed a tendency to aggregate, even at the uppermost temperatures, but all three exhibited a complete loss of binding activity at temperatures above their melting point (∼70°C). The behavior of the mAbs in particular demonstrates that the optical density measurements do not assess the percentage of folded material; binding assays are critical to determine function after heat denaturation.

### Improving thermal stability

**Figure 5:**
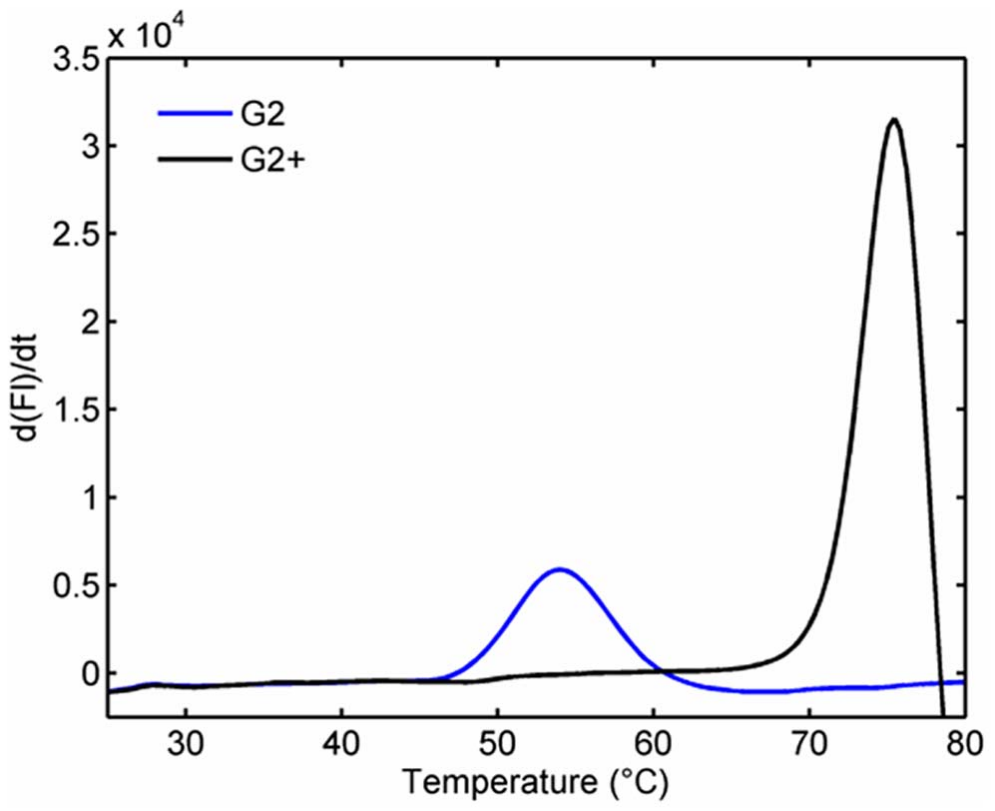
Increased melting temperature of L1-G2+. Fluorescence based melting assay showing melting of L1-G2 in blue and the mutant with the added disulfide, L1-G2+, in black. Melting temperature is determined from a plot of the first derivative of the melt curve. The measurements were performed in triplicate on two different days and showed identical results; for clarity only one replicate is shown.

The ability to refold and regain binding activity is a significant advantage of sdAbs compared to their conventional antibody counterparts. However, as seen in this work and others, not all sdAbs refold with the same efficiency [17], [18], [25]. Even sdAbs that show good refolding can be prone to aggregation when heated for extended time above their melting temperature; therefore it is desirable to engineer higher melting temperatures to ensure the best thermal stability. Improving stability of sdAb has been accomplished in a number of ways including through use of stringent selections [28], [29], [30], [31] or through the addition of disulfide bonds [32], [33]. In an attempt to increase the melting temperature of the sdAb L1-G2, cysteine residues were added in positions threonine 49 and leucine 70 (**Figure 1**) to facilitate the formation of a second disulfide bond between the framework 2 and 3 regions of the sdAb. The positions that we chose to mutate were analogous to those utilized by Hagihara et al. [33] who first showed increased melting temperature of sdAb with an introduced disulfide. Cysteine mutations at analogous positions have since been demonstrated to produce an increased melting temperature in sdAbs originating from llamas, alpacas and camels and appear to provide a universal method for increasing sdAb stability [32], [33], [34]. The resulting mutant was termed L1-G2+; as can be seen in **Table 3** and **Figure 5**, the addition of the disulfide bond increased the melting temperature of this particular sdAb by approximately 19°C. Protein preparations of the L1-G2+ mutant yielded around 3-4 mg sdAb per liter of bacterial expression culture; this was less than the parental L1-G2 which produced yields of ∼20 mg per liter using the same protocols.

The increased melting temperature of the L1-G2+ translated into improved activity at temperatures up to 75°C compared to the parental sdAb as seen in **Figure 4**, with the disulfide mutant retaining 100% of its activity at 75°C. Though the L1-G2+ mutant did show increased thermal stability, the added disulfide bond reduced the ability to refold following thermal denaturation in comparison to L1-G2. This can be seen by CD, **Figure S4 in** **File S1**. Also, in contrast to the parental sdAb, which retained approximately 20% of its binding ability after heating to 85°C, the disulfide mutant shows a complete loss of activity after identical heating. Other researchers have also observed that although addition of a non-canonical disulfide can lead to a substantial increase in Tm, it does not necessarily correlate with an increase in functional material when the protein is heated for an extended time above its Tm [35].

The additional disulfide bond also altered the binding kinetics of the L1-G2+ sdAb; however the slightly higher off rate appears to have been balanced by a faster on rate to result in effectively an unchanged K_D_. These changes in on and off rates may be due to a reduction in the flexibility of the molecule. Other researchers have reported mutants with additional disulfides achieved an increase of ∼10°C in stability [32], [33], but sometimes at the cost of a decreased affinity [32]. Adding a similarly placed disulfide was also reported to increase the protease resistance of sdAbs [34].

### Specificity of L1-binding sdAb

**Figure 6:**
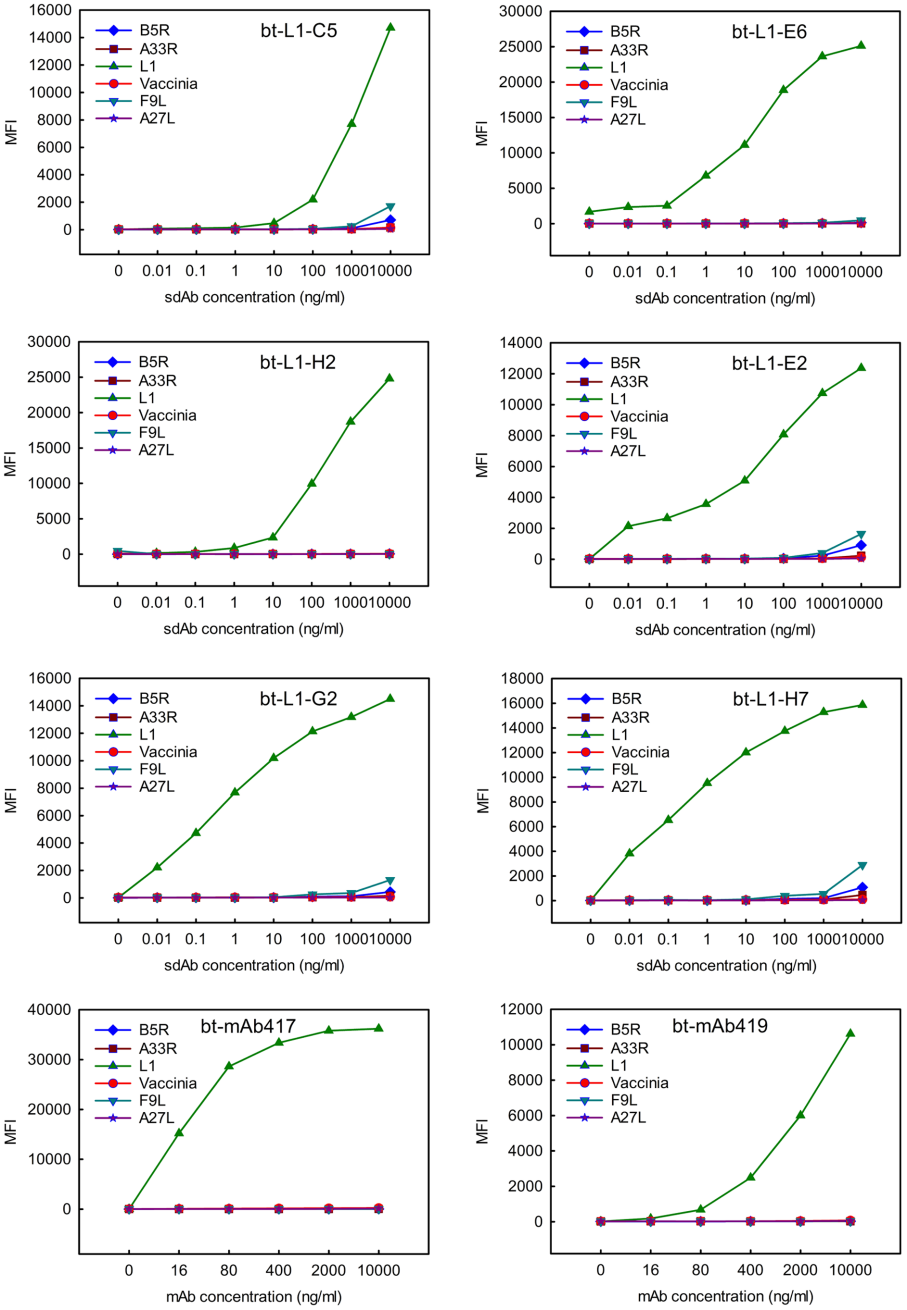
Specificity of L1 binding sdAbs. Direct binding assays were used to demonstrate target specificity for L1 binding antigens. Antigen was immobilized to magnetic beads and biotinylated (bt)- sdAbs (noted at the top of each graph) served as the tracers. Independent direct binding assays were performed different days; a representative data set is shown. The MAGPIX assays typically measures binding from at least 50 beads and the error of the mean fluorescence intensity (MFI) is ≤5%.

Direct binding assays utilizing the Luminex MAGPIX were employed to determine the specificity of the anti-L1 sdAb as the multiplexed format enabled the simultaneous assessment of binding of each sdAb to L1, four additional recombinant vaccinia proteins, and killed virus preparations. Each of the sdAbs isolated against L1 showed specificity for only the L1 antigen **Figure 6**. However, none of the L1 binders that were isolated and characterized were able to bind the killed vaccinia virus material. In addition, all three anti-L1 mAbs acquired through BEI resources were unable recognize the inactivated virus material in the bead-based assays. This was surprising as mAb417 was produced from the same hybridoma as mAb VMC2 which has been shown to inhibit the infectivity of the IMV indicating that the epitope of L1 recognized by this antibody should be accessible in the intact particle [5]. We speculate that that the lack of recognition of the vaccinia target by the sdAbs and mAbs in the MAGPIX assay may have more to do with the killed material used in the assays rather than with accessibility of the L1 epitope. The vaccinia virus was inactivated using the UV-psoralen method, which serves to crosslink the viral DNA [16]. As the psoralen reaction only involves the nucleic acids, the surface antigens should remain unmodified, so it is unclear why this material was not recognized [36], [37], [38]. The anti-L1 sdAbs and both mAbs also failed to recognize vaccinia virus that had been gamma irradiated; this was not as surprising as studies have reported a discrepancy in the ability of antibodies, including sdAbs, to recognize live versus irradiated virus [39], [40].

### Selection of sdAbs that recognize the vaccinia virus target

**Figure 7:**
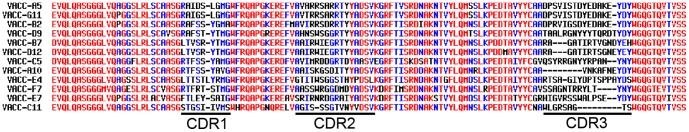
Amino acid alignments of vaccinia virus binding sdAbs. The representatives from each family that were chosen for further characterization are presented. Red denotes positions where the amino acid is 90% conserved in the compared sequences, blue indicates low consensus (50%) and black are not conserved. The three CDRs are underlined.

In an effort to isolate sdAbs that recognize the killed vaccinia, the library was panned against the viral target. Twenty six binding clones were sequenced after two rounds of selection against killed vaccinia virus and unique families were identified based on amino acid composition of the complementarity determining regions (CDRs). Sequences of the clones that were expressed for characterization are reported in **Figure 7**.

**Figure 8:**
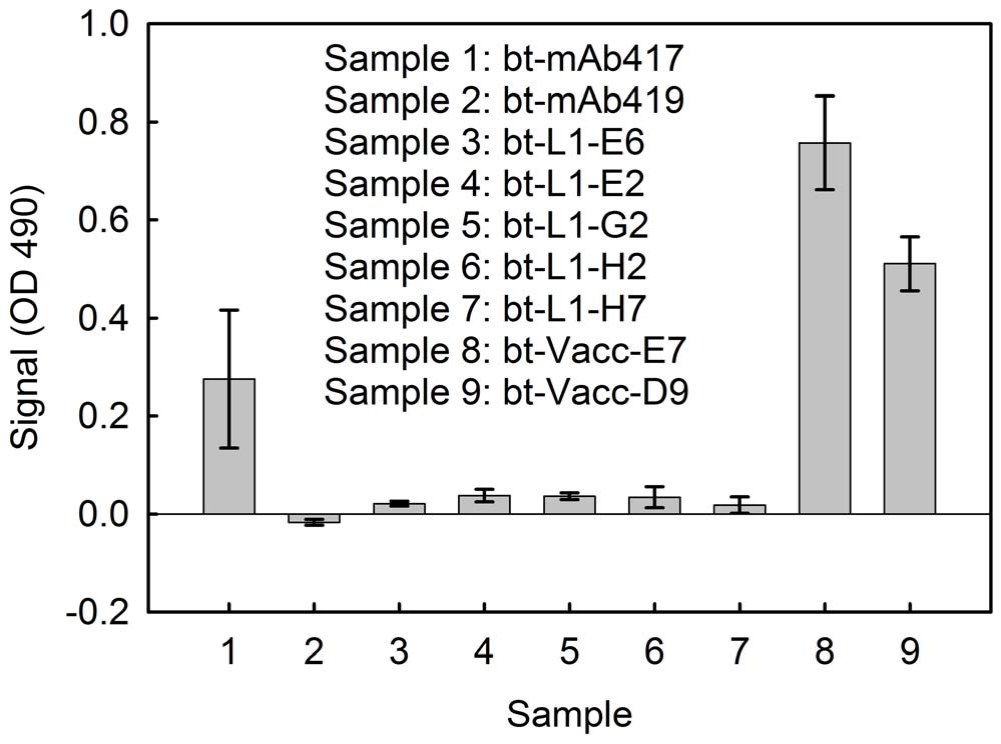
Direct binding ELISA to assess binding to inactivated vaccinia virus. The anti-L1 mAbs 417 and 419 as well as sdAb selected on L1 (L1-E6, L1-E2, L1-G2, L1-H2 and L1-H7) and sdAb selected on vaccinia (Vacc-E7 and Vacc-D9) were tested for binding to inactivated vaccinia virus immobilized on the wells of a 96-well plate. Measurements were done in triplicate; data is plotted as signal minus background, and the error bars represent the standard deviation.

The ability of select anti-vaccinia sdAbs, anti-L1 sdAbs and mAbs (mAb417 and mAb419) to recognize the vaccinia antigen was assessed via a direct binding ELISA that measured binding to passively immobilized virus (**Figure 8**). The sdAbs selected against vaccinia showed a signal to background ratio of at least 5. Neither mAb419 nor the anti-L1 sdAb showed any signal; while mAb417 showed a weak signal in this assay format of 3 times background.

Fluorescent based melting assays were employed to determine the melting temperatures of the anti-vaccinia sdAbs. The sdAbs showed a range of melting temperatures ranging from 57 to 79°C as reported in **Table 4**. This is typical of the values reported by both our group and others [22], [25].

**Table 4:** Melting temperatures of vaccinia binding sdAbs.

sdAb	Melting temperature (°C)*
Vacc-A5	60
Vacc-B7	79
Vacc-C5	57
Vacc-D9	71
Vacc-E7	60
Vacc-F7	62
Vacc-G11	61
Vacc-C11	59
Vacc-D12	75
Vacc-E4	70

* Fluorescent melt assays performed in triplicate; the measurements were typically identical within less than 0.2°C, and always within 0.6°C.

### Sandwich assay for vaccinia detection

**Figure 9:**
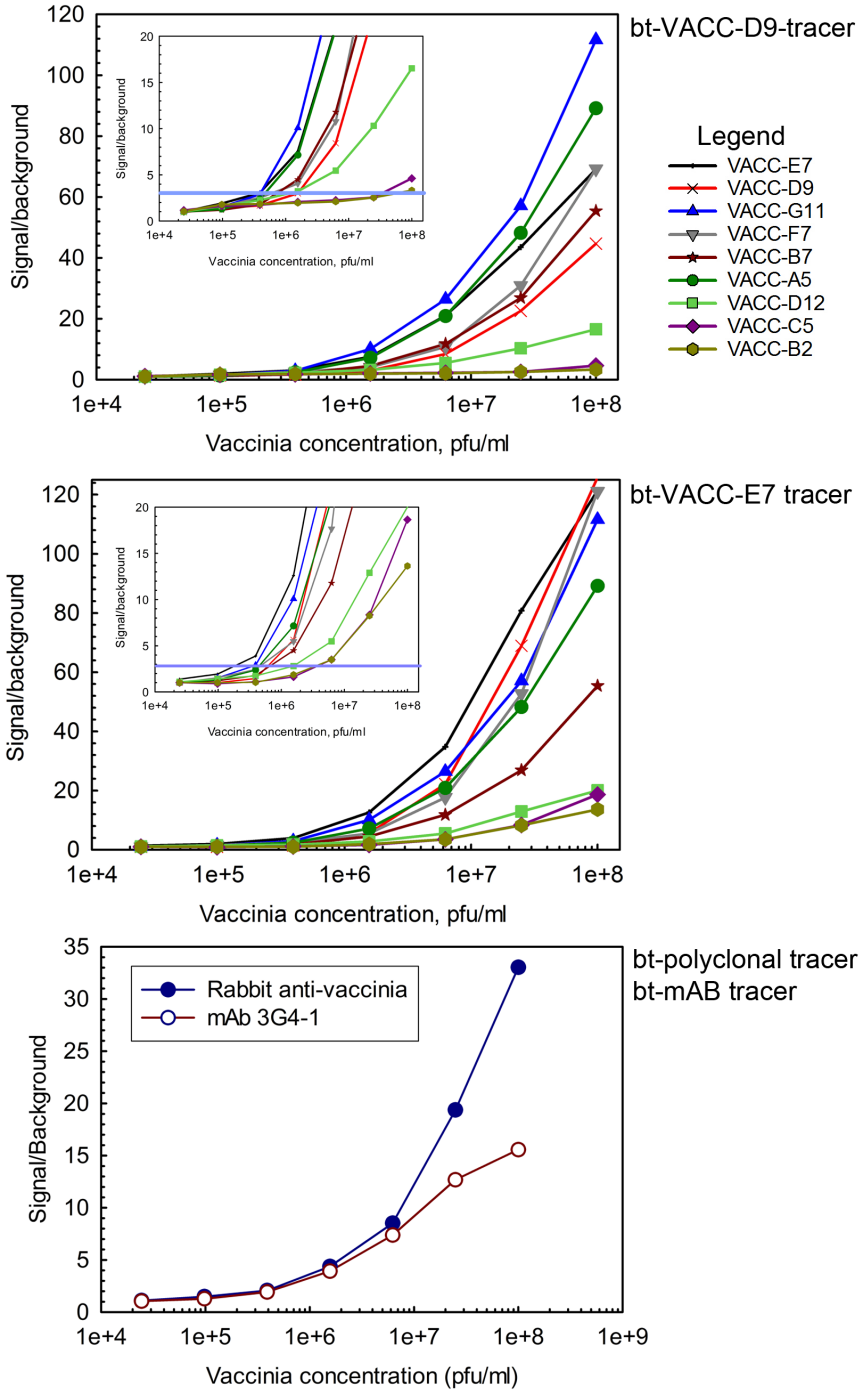
MAGPIX limit of detection assays for vaccinia virus. Top two panels show purified sdAbs used as both captures and tracers in bead-based assays to detect the vaccinia virus. The bottom panel shows the assay using conventional antibodies. Capture antibodies are plotted with corresponding biotinylated (Bt) tracer sdAbs indicated above each graph. The Y-axis is a measure of the signal over background while the X-axis represents the concentration of the vaccinia virus. Insets show a close-up of the lower part of the graph, the blue line indicates the LOD, defined as a signal to background ratio of 3. Limits of detection for best performing sdAb capture-tracer pairs approach 4×10^5^ pfu/ml. Sandwich assays for vaccinia detection were performed on multiple days and reproduced; a representative data set is shown. The MAGPIX assays measures binding from at least 50 beads and the error of the mean fluorescence intensity is within 5%.

The anti-vaccinia sdAbs were assayed as both captures and tracers in MAGPIX bead-based sandwich assays for the detection of vaccinia, **Figure 9** and **Figure S5 in File S1**. Limits of detection varied depending on the capture tracer combination used. A limit of detection (LOD) of 4×10^5^ pfu/ml was achieved with the best three combinations (VACC-G11 capture with VACC-D9 tracer, VACC-G11 capture with VACC- E7 tracer and VACC- E7 capture with VACC-E7 tracer). This LOD compares favorably with our results using conventional antibodies (1×10^6^ pfu/ml) (**Bottom panel** **figure 9**) and results reported by others obtained by a lateral flow assay (4 out of 7 at 1×10^6^ pfu/ml) [41].

None of the selected vaccinia binders were able to bind the recombinant L1 antigen in either a sandwich or direct binding format (data not shown). Given the complexity of the vaccinia virus, it was not surprising that the sdAbs selected on the viral target did not recognize the L1 antigen. The outer envelope and membranes on vaccinia virus are comprised of no fewer than 25 proteins with significant variability in protein content observed in the various infectious forms of the virus. Four additional recombinant vaccinia proteins (A27, A33, B5, and F9) were obtained from BEI Resources and included in the bead based assays; as with L1, none were recognized by the sdAbs isolated against the killed virus. Interestingly, the sdAbs selected against vaccinia bound well to the virus inactivated by the UV-psoralen method, but showed reduced binding to the gamma irradiated vaccinia.

## Conclusions

We successfully isolated sdAbs from an immunized llama library capable of binding either recombinant L1 protein or killed vaccinia virus Lister strain. Characterization of the L1 binding sdAbs showed nanomolar binding kinetics. A simple modification to the amino acid composition, the addition of cysteines to facilitate disulfide bond formation, had a dramatic effect on the thermal stability of a representative L1-binding sdAb, raising its melting temperature nearly 20°C.

Unfortunately, the L1 binding sdAbs were not able to bind the killed vaccinia antigen. The initial immunizations with the viral particle would have produced antibodies against many of the viral proteins. The inability of the L1 binding sdAbs to bind the viral target was unexpected. It was anticipated that immunizing with both the viral particle and boosting with the recombinant antigen would have generated a strong immune response for the L1 antigen both as a viral envelope protein and as a free antigen. The poor virus recognition of the conventional antibody mAb417 and lack of recognition by mAb419, which share binding epitopes with our sdAbs, suggests some deficiency in the sample used for these studies. The vaccinia virus replication cycle and diversity of strains could contribute to variability in virus samples. Future studies assaying live virus as well as killed material from several sources and strains may show better binding capabilities of the anti-L1 sdAbs for the intact viral particle.

Selections on vaccinia provided useful reagents that showed improved limits of detection versus conventional antibodies. SdAbs continue to gain momentum for use as both diagnostics and therapeutics. Not only do these molecules demonstrate excellent affinities, specificities, and thermal stability but also characteristics such as ease of production and engineering making sdAb attractive alternatives to conventional mono or polyclonal antibodies.

## Supporting Information

File S1 **File S1 contains figures showing**: Titer of plasma on killed vaccinia and recombinant L1 antigen; Phage from individual clones from the initial L1 selection binding to bead-immobilized L1; Surface plasmon resonance data for the L1 binding sdAb; Refolding of the L1 sdAb assessed by circular dichroism; Magplex sandwich assays for limit of detection (vaccinia) using different capture and tracer pairs.
(DOC)
