# Supplementary material for: Development and Evaluation of Single Domain Antibodies for Vaccinia and the L1 Antigen
Source: PLoS One. 2014 Sep 11;9(9):e106263. doi: 10.1371/journal.pone.0106263 (PMC4161341; doi:10.1371/journal.pone.0106263)

**Supplemental Information**

**Development and Evaluation of Single Domain Antibodies for Vaccinia and the L1 Antigen**

Scott A. Walper, Jinny L. Liu, Daniel Zabetakis, George P. Anderson, and Ellen R. Goldman*

Naval Research Laboratory, Washington, DC USA

*Corresponding Author: [ellen.goldman@nrl.navy.mil](mailto:ellen.goldman@nrl.navy.mil), Fax: 202-767-9594

Supplemental Information

Contents:

S-1. Title page

S-2. **Supplemental figure S1.** Titer of plasma on killed vaccinia and recombinant L1 antigen

S-3. **Supplemental figure S2.** Phage from individual clones from the initial L1 selection binding to bead-immobilized L1

S-4. **Supplemental figure S3.** Surface plasmon resonance data for the L1 binding sdAb

S-5. **Supplemental figure S4.** Refolding of L1 sdAb assessed by Circular dichroism.

S-7. **Supplemental figure S5.** Magplex sandwich assays for limit of detection (vaccinia)

**Figure S1 .**  Titer of plasma on killed vaccinia material, recombinant L1 antigen, and BSA. Top graphs are results after immunization with the vaccinia antigen. Bottom panels are after two boosts with the recombinant L1 antigen. Measurements were performed in triplicate, error bars represent the standard deviation.

**Figure S2** . Initial selection of L1 binding sdAb. Phage from individual clones were used in bead-based assays with mAb captures to identify those capable of binding the L1 antigen. An anti-M13 mAb labeled with phycoerythin served as the secondary antibody for determining mean fluorescence intensity (MFI; y-axis)

**
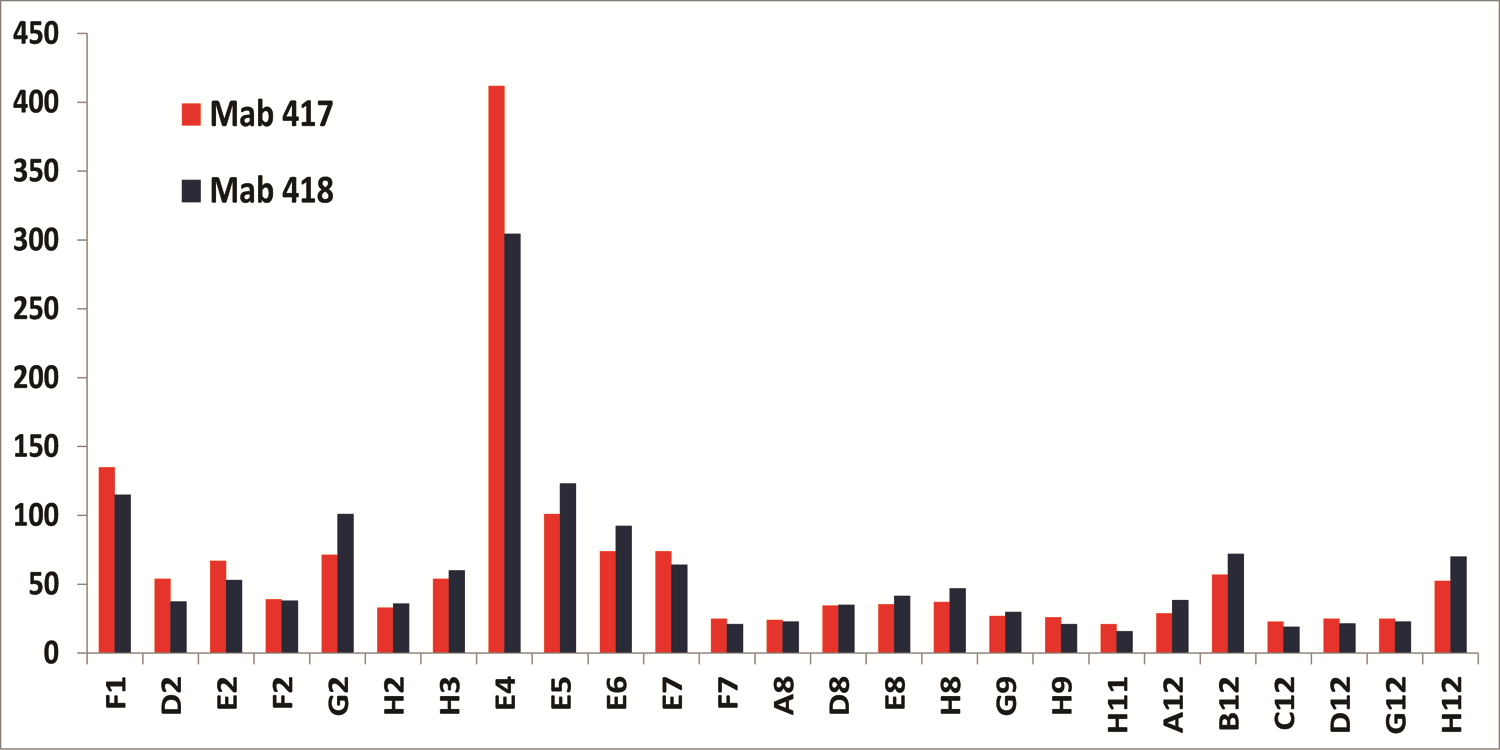
**

**Figure S3**. Surface Plasmon resonance data for the L1 binding sdAbs (representative data). The kinetics reported in Table 1 of the manuscript were derived from the fits of the average of three data sets. Each data set included fits from4 or 5 concentrations of the sdAbs (from 3.7 to 300 nM) binding to L1 coated surfaces, as shown below. All three sets were practically identical. Standard deviations are given in Table 1 of the manuscript.

**
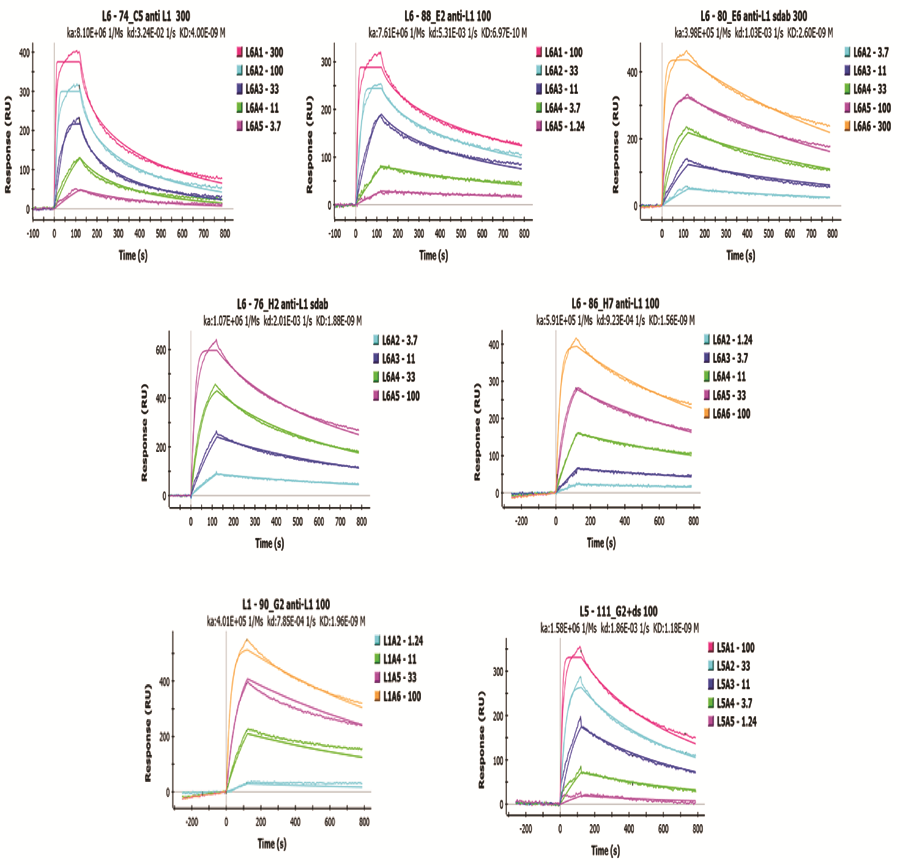
**

**Figure S4.** Circular dichroism to assess the refolding properties of each of the sdAbs targeting the L1 antigen. Temperature is expressed in °C and change in ellipticity is in milidegrees. Panel A shows the spectra of clone L1-H7 before heating (blue curve), when denatured at 75 °C (red curve) and again at room temperature after cooling (green curve). This shows how L1-H7 retains its secondary structure after heating. Panel B. Melting and re-heating curves of the sdAb clones. Each sdAb was heated and cooled two times. Elipticity is monitored at a constant wavelength (208 nm) as the temperature was increased and decreased.

A.


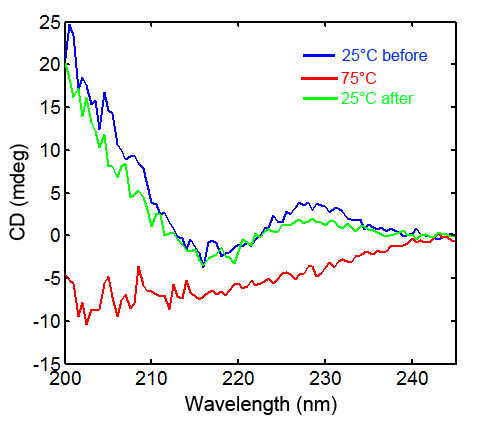


B.

**
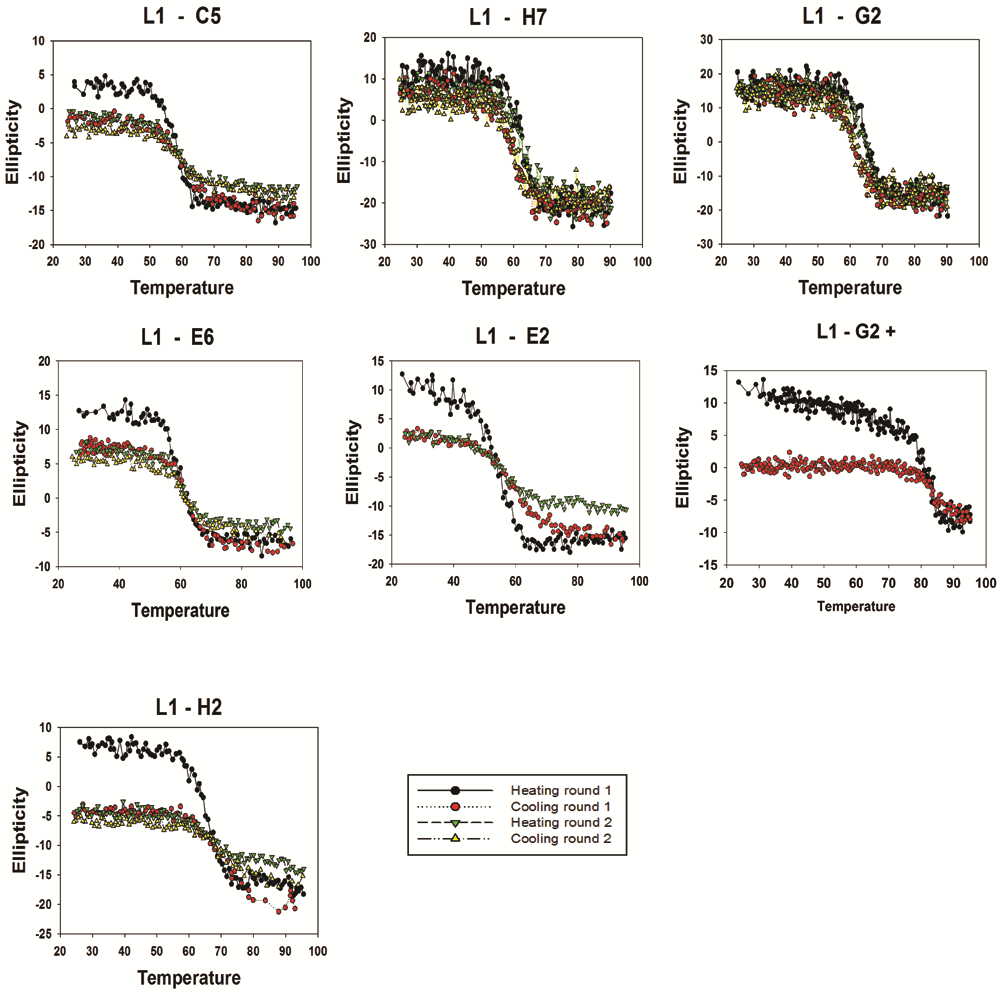
**

**Figure S5.** Magplex sandwich assays for limit of detection. Panel A shows data with a variety of sdAb capture paired with a panel of tracers. SdAbs selected for the viral particle were assayed as both biotinylated (bt) tracers (graph label) and captors (legend) in limit of detection assays. B. Data from highlighted assays with bt-VACC-D9 and bt-VACC-E7 tracers and sdAb captures. Data is shown both as the logarithmic and linear plot to allow for better visualization of the lower antigen concentrations. A ratio of signal over background of 3 was considered positive. Limits of detection for the two best performing sdAbs approaches 4x10^5^ pfu/ml.

**A**


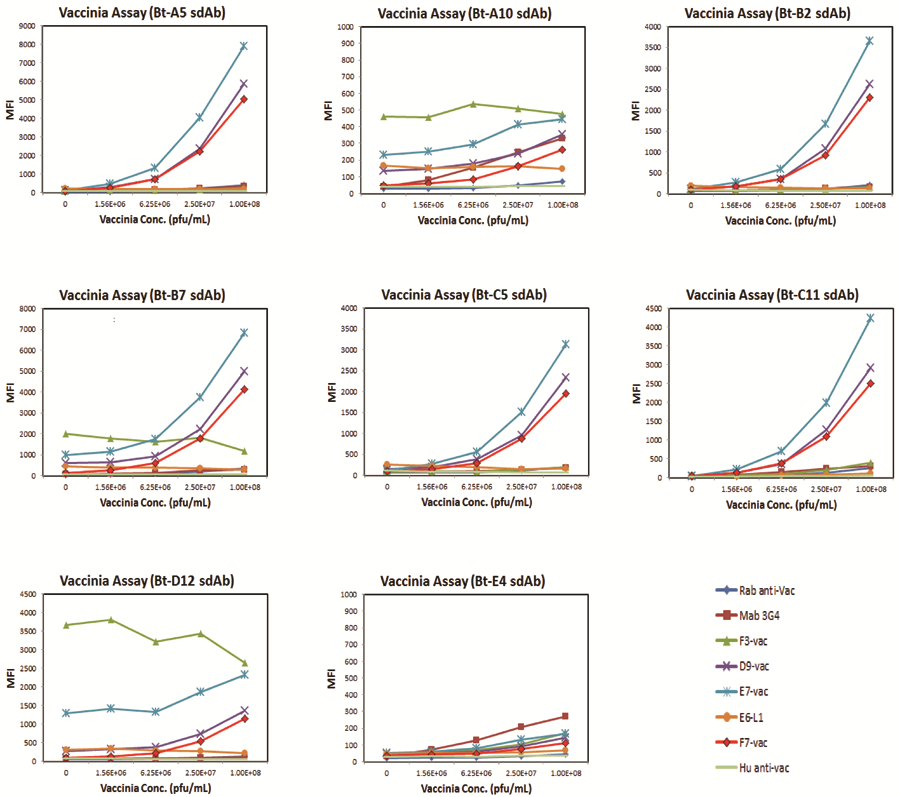


**B**


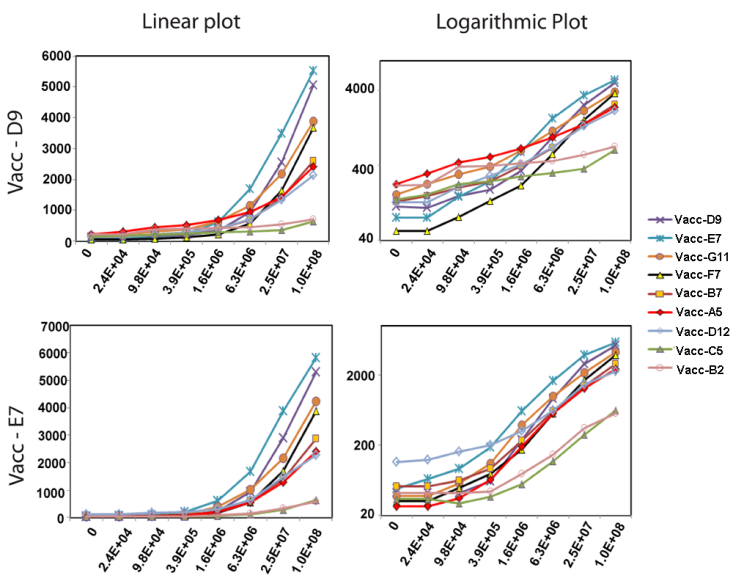

Supplement: File S1 — contains figures showing: Titer of plasma on killed vaccinia and recombinant L1 antigen; Phage from individual clones from the initial L1 selection binding to bead-immobilized L1; Surface plasmon resonance data for the L1 binding sdAb; Refolding of the L1 sdAb assessed by circular dichroism; Magplex sandwich assays for limit of detection (vaccinia) using different capture and tracer pairs. (DOC) [file pone.0106263.s001.doc]
